# SPRIN_2_D nomogram: predicting 90-day unfavorable outcomes in patients with minor ischemic stroke receiving intravenous thrombolysis

**DOI:** 10.3389/fneur.2026.1841196

**Published:** 2026-08-03

**Authors:** Jiao Ye, Yan Wan, Yifan Zhou, Qinghui Xiao, Qingcan Li, Wenyong Gao, Jichuan Hu, Ming Huang, Bo Hu, Huijuan Jin

**Affiliations:** 1 Department of Neurology, Union Hospital, Tongji Medical College, Huazhong University of Science and Technology, Wuhan, China; 2 Department of Neurology, Wuhan Hankou Hospital, Wuhan, China; 3 Department of Neurology, People’s Hospital of Dongxihu District, Wuhan, China; 4 Department of Neurology, Hubei Provincial Hospital of Integrated Chinese and Western Medicine, Hubei University of Chinese Medicine, Wuhan, China

**Keywords:** intravenous thrombolysis, minor ischemic stroke, nomogram, prediction model, prognosis

## Abstract

**Objective:**

This study aimed to construct and validate a nomogram for the intuitive prediction of 90-day unfavorable outcomes in patients with minor ischemic stroke who underwent intravenous thrombolysis.

**Methods:**

Data were extracted from the Multicenter Clinical Trial of Revascularization Treatment for Acute Ischemic Stroke (TRAIS) study. Multivariable logistic regression was used to identify independent factors associated with 90-day unfavorable outcomes (modified Rankin Scale [mRS] score 2–6) in patients with minor ischemic stroke (National Institutes of Health Stroke Scale [NIHSS] score 0–5) receiving intravenous thrombolysis. A nomogram (designated as SPRIN_2_D) was developed based on the final prediction model, incorporating 6 key variables: NIHSS score, functional disability, systolic blood pressure, neutrophil count, international normalized ratio and renal dysfunction.

**Results:**

A total of 1,438 eligible patients were enrolled and randomly assigned to a derivation cohort (*n* = 1,007) and a validation cohort (*n* = 431). In the overall cohort, the median age was 65 years (interquartile range, 56–73); 67.94% of participants were male, and the median admission NIHSS score of 2 (interquartile range, 1–4). The SPRIN_2_D nomogram exhibited robust performance in predicting 90-day unfavorable outcomes in minor ischemic stroke patients treated with intravenous thrombolysis. The area under the curve was 0.784 (95% confidence interval [CI], 0.744–0.822) in the derivation cohort and 0.776 (95% CI, 0.716–0.835) in the validation cohort. Moreover, the SPRIN_2_D nomogram demonstrated superior discriminative ability compared with other existing prognostic models for acute ischemic stroke and minor ischemic stroke.

**Conclusion:**

The SPRIN_2_D nomogram is a stable and promising tool for predicting 90-day unfavorable outcomes in minor ischemic stroke patients treated with intravenous thrombolysis.

## 1 Introduction

Acute ischemic stroke (AIS) remains a leading global cause of disability and mortality (1). Despite significant advancements in diagnostic and therapeutic strategies, the associated social and economic burdens continue to escalate (2). Minor ischemic stroke (MIS) accounts for approximately 50% of all patients with AIS (3, 4). Notably, the prognosis of MIS patients is not optimistic: 13.6% experience early neurological deterioration (END), which significantly increases the risk of stroke recurrence, disability, and death, highlighting the necessity of proactive clinical management (5, 6).

Intravenous thrombolysis (IVT) is the guideline-recommended first-line treatment for AIS, but its efficacy is highly time-dependent, requiring administration within a 4.5 h therapeutic window. However, IVT utilization among MIS patients remains suboptimal: fewer than half of MIS patients in the United States receive this therapy, with even lower rates reported in China (7, 8). This underutilization stems from clinical uncertainty. Current guidelines recommend IVT for MIS patients with disabling symptoms, but lack sufficient evidence to support its use in non-disabling cases (9). Importantly,a diagnosis of MIS does not guarantee a favorable outcome; patients with low National Institutes of Health Stroke Scale (NIHSS) scores may still develop subsequent functional impairments (10). For instance, studies have indicated that approximately one-third of MIS patients without IVT treatment have an unfavorable outcome at 3 months (11), while several clinical trials have demonstrated the potential benefits of IVT in this population (12). Thus, relying solely on neurological deficit types and NIHSS scores to guide thrombolysis decisions for MIS patients is overly restrictive. There is an urgent need to establish an integrated predictive model for unfavorable outcomes in IVT-treated MIS patients.

Previous studies have identified multiple factors associated with unfavorable outcomes in MIS patients undergoing IVT, including stroke severity, NIHSS score, age, sex, diabetes mellitus, and hypertension (13, 14). However, existing thrombolysis-related prognostic models lack a specific focus on MIS patients (15, 16). To address this gap, the present study aims to develop a stable nomogram for predicting the risk of unfavorable outcomes in MIS patients who received IVT therapy. By integrating key clinical, demographic, and laboratory variables, this tool aims to provide clinicians with an evidence-based approach for identifying patients at high risk of unfavorable outcomes at an early stage, thereby optimizing the efficient utilization of medical resources and improving patient outcomes.

## 2 Materials and methods

### 2.1 Study population

The data for this study were derived from the Multicenter Clinical Trial of Revascularization Treatment for Acute Ischemic Stroke (TRAIS) study, a multicenter cohort that enrolled patients from 14 hospital centers between June 2020 and January 2025 (17). Patients were recruited in accordance with the following inclusion criteria: (1) received IVT within the time window according to thrombolytic therapy indications; (2) clinical and radiological diagnosis of AIS; (3) age ≥18 years; Exclusion criteria were as follows: (1) cerebral hemorrhage or contraindications to thrombolytic therapy; (2) incomplete clinical data; (3) mental disorders or severe cognitive impairment. Patients with NIHSS scores ≤ 5 were included in the study.

This study was approved by the Ethics Committee of Union Hospital, Tongji Medical College, Huazhong University of Science and Technology, registered in the Chinese Clinical Trial Registry (ChiCTR2000033456), and written informed consent was obtained from all participants prior to enrollment.

### 2.2 Collection of clinical data

Demographic characteristics (age and gender) and medical history information were collected from all patients at admission. Hypertension was defined as meeting one or more of the following criteria: blood pressure ≥ 140/90 mmHg, self-reported physician-diagnosed hypertension, or current use of antihypertensive medications (18). Hyperlipidemia was identified as elevated blood lipid levels (total cholesterol ≥ 6.22 mmol/L, triglyceride ≥ 2.26 mmol/L, low-density lipoprotein cholesterol ≥ 4.14 mmol/L) or a documented history of hyperlipidemia (19). Diabetes was defined as a fasting blood glucose (FBG) level ≥ 7.0 mmol/L, physician-diagnosed diabetes, or current use of antidiabetic drugs. Renal dysfunction was characterized by the presence of albuminuria (urine albumin-creatinine ratio ≥ 30 mg/g), abnormal urine sediment, renal tubule-related lesions, histological or structural renal abnormalities, history of kidney transplantation, reduced glomerular filtration rate (GFR < 60 mL/min/1.73 m^2^), or self-reported chronic kidney disease (20).

Admission clinical characteristics, including baseline systolic blood pressure (SBP), baseline diastolic blood pressure (DBP), NIHSS score, and functional disability, were assessed by experienced neurologists before IVT treatment. Functional disability was assessed before IVT administration using baseline NIHSS item scores. Patients were classified as having functional disability if they presented with any of the following potentially disabling neurological deficits: complete hemianopia (NIHSS score ≥ 2), severe aphasia (NIHSS score ≥ 2), neglect (NIHSS score ≥ 1), or limb weakness (NIHSS score ≥ 2) (21).

Laboratory parameters, including lymphocyte count, neutrophil count, platelet count, prothrombin time (PT), fibrinogen, activated partial thromboplastin time (APTT) and international normalized ratio (INR), were collected on admission. FBG was measured within 12 h after enrollment.

### 2.3 Outcome

The primary outcome was defined as an unfavorable outcome, which was identified as a modified Rankin Scale (mRS) score of 2–6 at 90 days post-treatment. This cutoff was selected given that an mRS score of 2 or higher is the widely accepted standard for identifying clinically relevant long-term functional dependence after AIS. The mRS score was assessed through telephone interviews or electronic communication with patients or their authorized representatives and all assessors were blinded to patients’ baseline clinical characteristics to ensure consistent and reliable outcome collection.

### 2.4 Statistical analysis

The Kolmogorov–Smirnov test was applied to assess the normality of data distribution. Continuous variables were expressed as the mean ± standard deviation (SD) if normally distributed or median with interquartile range (IQR) if non-normally distributed. Categorical variables were presented as frequencies (%) and analyzed using chi-square tests. Differences between groups were examined by independent samples t-tests (for normally distributed data) or Mann–Whitney U tests (for non-normally distributed data), with a two-sided *p* < 0.05 considered statistically significant. Intra-group differences were analyzed using independent sample two-tailed t-tests or Mann–Whitney U tests, as appropriate.

Logistic regression analyses were conducted to identify potential prognostic predictors, with results presented as odds ratios (ORs) and corresponding 95% confidence interval (CI). Variables with *p* < 0.05 in the univariate logistic analysis were incorporated into stepwise multivariate logistic regression to screen for independent prognostic factors. The discriminative ability of the nomogram was evaluated using the receiver operating characteristic (ROC) curves, with the area under the curve (AUC) serving as the key indicator.

Patients were randomly divided into the derivation cohort and validation cohort at a ratio of 7:3. Univariate and multivariate logistic regression analyses were conducted in the derivation cohort to construct the SPRIN_2_D nomogram, which was then validated in both cohorts. Each predictor was assigned a weight based on its regression coefficient. For each individual patient, the weighted sum of all predictors, together with the model intercept, was entered into the logistic function to calculate the estimated probability of a 90-day unfavorable outcome.

The “rms” and “rmda” packages in R software were used to construct the nomogram and perform clinical decision curve analysis (DCA). Calibration curves were plotted to assess the consistency between the predicted probabilities and the observed outcomes of the logistic regression model. To evaluate the predictive performance of the nomogram for 90-day unfavorable outcomes, we calculated diagnostic indices including sensitivity, specificity, positive predictive value (PPV), negative predictive value (NPV), positive likelihood ratio (PLR) and negative likelihood ratio (NLR) at distinct candidate score thresholds. Subsequently, we defined the risk stratification cutoffs in a data-driven manner by comprehensively analyzing the distribution of 90-day unfavorable outcome rates across nomogram scores, variations in diagnostic indices at different threshold values, and the clinical applicability of the stratified groups. Using these optimized score cutoffs, patients were categorized into low-, medium-, and high-risk groups. Cutoff-based risk estimates were separately calculated in the derivation and validation cohorts, and the incidence of 90-day unfavorable outcomes was calculated for each risk group. A linear trend test was performed to assess the stepwise increase in risk across the three strata.

Furthermore, we compared the discriminative ability of the SPRIN_2_D nomogram with existing prognostic models for AIS and MIS model in predicting 90-day unfavorable outcomes (mRS score ≥ 2) in the validation cohort. For a more comprehensive comparison, calibration performance and clinical utility were also assessed for all included models. All statistical analyses were performed using R software (version 4.3.2) and SPSS statistics (version 29.0.2).

## 3 Result

### 3.1 Baseline characteristics of participants

**Figure 1:**
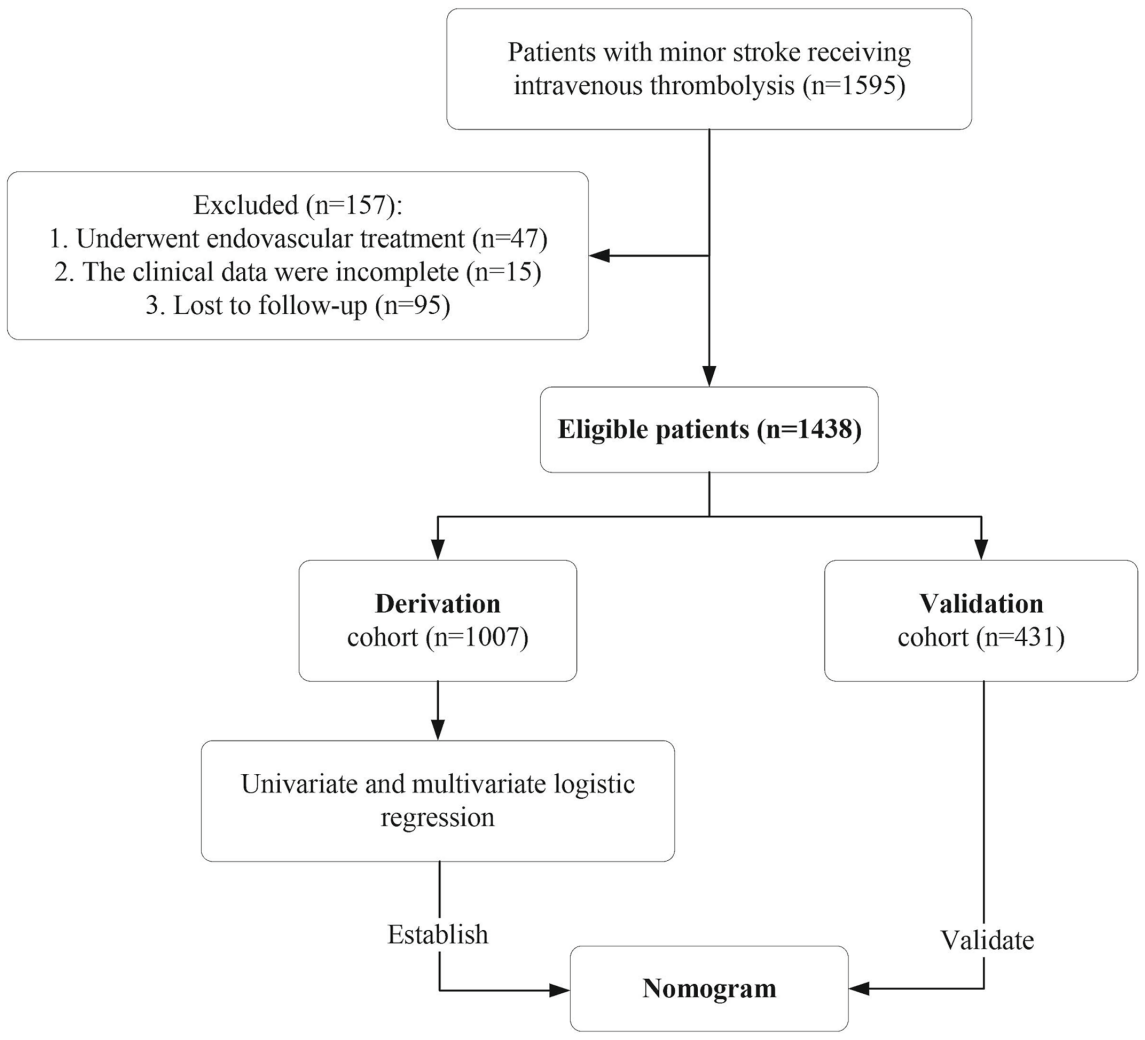
The flowchart of study population.

A total of 2,336 participants were enrolled in the TRAIS study, among whom 1,595 were diagnosed with MIS. Patients were excluded if they had incomplete clinical data (*n* = 15), were lost to follow-up (*n* = 95), or underwent endovascular treatment (*n* = 47). Incomplete clinical data were mainly due to the absence of subsequent examinations or further treatment after thrombolysis. The term “lost to follow-up” was defined as the unavailability of 90-day mRS scores. After screening, 1,438 eligible MIS patients were randomly assigned to the derivation cohort (*n* = 1,007) and the validation cohort (*n* = 431) at a 7:3 ratio (Figure 1). Unfavorable outcomes (mRS score ≥ 2) were observed in 167 patients (16.58%) in the derivation cohort and 56 patients (12.99%) in the validation cohort.

Baseline characteristics of the participants, including demographic data, clinical characteristics, and laboratory measurements, are summarized in Table 1. The median age of the total cohort was 65 (IQR, 56–73) years, 67.94% were male, and the median baseline NIHSS score was 2 (IQR, 1–4). No statistically significant differences were observed in any baseline characteristics between the two cohorts (*p* = 0.072–0.994) confirming the effectiveness of random allocation (Table 1).

**Table 1:** Baseline characteristics of participants stratified by derivation and validation cohorts.

Characteristics	Total (*n* = 1,438)	Derivation cohort (*n* = 1,007)	Validation cohort (*n* = 431)	*p* value
Demographic characteristics				
Age, M (Q₁, Q₃)	65 (56, 73)	65 (56, 73)	64 (56.5, 72)	0.994
Gender [Male, n (%)]	977 (67.94)	693 (68.82)	284 (65.89)	0.276
Medical history, n (%)				
Smoking	426 (29.62)	302 (29.99)	124 (28.77)	0.643
Alcohol	274 (19.05)	201 (19.96)	73 (16.94)	0.181
Ischemic stroke	203 (14.12)	142 (14.10)	61 (14.15)	0.979
Intracranial hemorrhage	22 (1.53)	15 (1.49)	7 (1.62)	0.849
Hypertension	840 (58.41)	580 (57.60)	260 (60.32)	0.336
Diabetes mellitus	364 (25.31)	249 (24.73)	115 (26.68)	0.435
Hyperlipemia	255 (17.73)	173 (17.18)	82 (19.03)	0.401
Coronary heart disease	179 (12.45)	122 (12.12)	57 (13.23)	0.559
Atrial fibrillation	84 (5.84)	56 (5.56)	28 (6.50)	0.488
Thyroid disorder	63 (4.38)	45 (4.47)	18 (4.18)	0.804
Renal dysfunction	133 (9.25)	102 (10.13)	31 (7.17)	0.078
Patent foramen ovale	15 (1.04)	13 (1.29)	2 (0.46)	0.258
Valvulopathy	37 (2.57)	30 (2.98)	7 (1.62)	0.137
Admission characteristics, M (Q₁, Q₃)				
NIHSS score	2 (1, 4)	2 (1, 4)	3 (1, 4)	0.324
SBP, mmHg	146 (132, 159)	146 (133, 159)	146 (132, 160)	0.495
DBP, mmHg	84 (76, 92)	84 (75, 91)	84 (77, 92)	0.340
Functional Disability	414 (28.79)	280 (27.81)	134 (31.09)	0.207
Admission laboratory values, M (Q₁, Q₃)				
Leukocyte count, ×10^9^/L	6.76 (5.54, 8.51)	6.73 (5.54, 8.4)	6.80 (5.57, 8.70)	0.482
Erythrocyte count, ×10^12^/L	4.52 (4.18, 4.85)	4.51 (4.17, 4.85)	4.56 (4.25, 4.85)	0.201
Platelet count, ×10^9^/L	201 (169, 239)	200 (168, 239)	204 (171, 241)	0.502
Neutrophil count, ×10^9^/L	4.56 (3.50, 6.27)	4.57 (3.53, 6.17)	4.54 (3.44, 6.45)	0.986
Lymphocyte count, ×10^9^/L	1.48 (1.06, 2.00)	1.47 (1.04, 2.00)	1.50 (1.12, 2.01)	0.220
INR	0.99 (0.94, 1.04)	0.99 (0.94, 1.04)	0.98 (0.94, 1.03)	0.122
PT, s	12.60 (11.80, 13.20)	12.6 (11.80, 13.20)	12.5 (11.60, 13.10)	0.072
APTT, s	33.30 (30.7, 36.08)	33.40 (30.9, 36.1)	33.10 (30.55, 35.65)	0.186
FIB, g/L	3.19 (2.75, 3.68)	3.19 (2.75, 3.67)	3.21 (2.75, 3.69)	0.874
FBG, mmol/L	6.40 (5.40, 8.10)	6.40 (5.40, 8.10)	6.32 (5.36, 7.93)	0.894
Total bilirubin, μmol/L	10.50 (7.70, 14.3)	10.3 (7.70, 14.15)	10.9 (7.60, 14.40)	0.306
Direct bilirubin, μmol/L	3.30 (2.30, 4.70)	3.40 (2.40, 4.70)	3.20 (2.20, 4.85)	0.711
Indirect bilirubin, μmol/L	7.10 (5.00, 9.90)	7.00 (4.90, 9.80)	7.6 (5.00, 10.29)	0.089

Continuous variables were described using mean ± SD or median (IQR), and categorical variables were indicated as n (%). NIHSS, National Institutes of Health Stroke Scale; SBP, systolic blood pressure; DBP, diastolic blood pressure; INR, international normalized ratio; PT, prothrombin time; APTT, activated partial thromboplastin time; FIB, fibrinogen; FBG, fasting blood glucose.

### 3.2 Univariate and multivariate analyses

In the derivation cohort, univariate logistic regression analysis indicated that patients with unfavorable outcomes had significantly higher SBP (*p* < 0.001), DBP (*p* < 0.001), FBG levels (*p* < 0.001), neutrophil counts (*p* = 0.004), INR (*p* < 0.001) and NIHSS scores (*p* < 0.001) compared with those with favorable outcomes. Additionally, medical histories of renal dysfunction (*p* < 0.001) and hypertension (*p* = 0.018) were more prevalent in MIS patients with unfavorable outcomes (Table 2). We subsequently conducted multicollinearity analysis for the above variables, and all variance inflation factor (VIF) values were below 5, indicating the absence of severe multicollinearity among these variables (Supplementary Table S1).

**Table 2:** Univariate logistic regression analysis for 90-day outcomes in the derivation cohort.

Characteristics	Favorable outcome (*n* = 840)	Unfavorable outcome (*n* = 167)	*p* Value
Demographic characteristics			
Age	64 (56, 72)	66 (56.5, 76)	0.123
Gender			0.267
Female	268 (31.90)	46 (27.54)	
Male	272 (68.10)	121 (72.46)	
Medical history, n (%)			
Smoking			0.700
No	586 (69.76)	119 (71.26)	
Yes	254 (30.24)	48 (28.74)	
Alcohol			0.437
No	676 (80.48)	130 (77.84)	
Yes	164 (19.52)	37 (22.16)	
Ischemic stroke			0.279
No	726 (86.43)	139 (83.23)	
Yes	114 (13.57)	28 (16.77)	
Intracranial hemorrhage			0.479
No	829 (98.69)	163 (97.60)	
Yes	11 (1.31)	4 (2.40)	
Hypertension			0.018^*^
No	370 (44.05)	57 (34.13)	
Yes	470 (55.95)	110 (65.87)	
Diabetes mellitus			0.355
No	637 (75.83)	121 (72.46)	
Yes	203 (24.17)	46 (27.54)	
Hyperlipemia			0.407
No	692 (82.38)	142 (85.03)	
Yes	148 (17.62)	25 (14.97)	
Coronary heart disease			0.562
No	736 (87.62)	149 (89.22)	
Yes	104 (12.38)	18 (10.78)	
Atrial fibrillation			0.316
No	796 (94.76)	155 (92.81)	
Yes	44 (5.24)	12 (7.19)	
Thyroid disorder			0.849
No	802 (95.48)	160 (95.81)	
Yes	38 (4.52)	7 (4.19)	
Renal dysfunction			<0.001^*^
No	779 (92.74)	126 (75.45)	
Yes	61 (7.26)	41 (24.55)	
Patent foramen ovale			0.623
No	828 (98.57)	166 (99.40)	
Yes	12 (1.43)	1 (0.60)	
Valvulopathy			0.462
No	813 (96.79)	164 (98.20)	
Yes	27 (3.21)	3 (1.80)	
Admission characteristics, M (Q₁, Q₃)			
NIHSS score	2 (1, 3)	4 (2, 4.5)	<0.001^*^
SBP, mmHg	144 (131, 156)	153 (142, 165)	<0.001^*^
DBP, mmHg	83 (75, 90)	88 (78, 98)	<0.001^*^
Functional Disability	195 (23.21)	85 (50.90)	<0.001^*^
Admission laboratory values, M (Q₁, Q₃)			
Leukocyte count, ×10^9^/L	6.71 (5.54, 8.19)	7.20 (5.54, 8.89)	0.163
Erythrocyte count, ×10^9^/L	4.51 (4.18, 4.84)	4.49 (4.08, 4.87)	0.430
Platelet count, ×10^9^/L	199.5 (169, 238)	203 (167.5, 243)	0.486
Neutrophil count, ×10^9^/L	4.51 (3.50, 5.90)	4.96 (3.74, 6.96)	0.004^*^
Lymphocyte count, ×10^9^/L	1.48 (1.06, 2.00)	1.41 (0.99, 1.95)	0.136
INR	0.99 (0.94, 1.03)	1.01 (0.97, 1.07)	<0.001^*^
PT, s	12.60 (11.90, 13.20)	12.60 (11.70, 13.40)	0.990
APTT, s	33.40 (31.08, 36.10)	33.00 (30.20, 36.10)	0.278
FIB, g/L	3.17 (2.76, 3.65)	3.27 (2.75, 3.73)	0.699
FBG, mmol/L	6.29 (5.3, 7.81)	6.80 (5.80, 9.06)	<0.001^*^
Total bilirubin, μmol/L	10.30 (7.68, 13.90)	10.40 (7.70, 15.20)	0.169
Direct bilirubin, μmol/L	3.30 (2.30, 4.62)	3.50 (2.45, 4.75)	0.377
Indirect bilirubin, μmol/L	6.90 (5.00, 9.50)	7.20 (4.75, 10.65)	0.212

Categorical variables were indicated as n (%). NIHSS, National Institutes of Health Stroke Scale; SBP, systolic blood pressure; DBP, diastolic blood pressure; INR, international normalized ratio; PT, prothrombin time; APTT, activated partial thromboplastin time; FIB, fibrinogen; FBG, fasting blood glucose. *p < 0.05.

For multivariate analysis, continuous variables were binarized using clinically relevant cutoffs determined by routine clinical practice and laboratory reference ranges. All cutoffs were rounded to the nearest integer to enhance clinical feasibility and applicability. Given the limited range of NIHSS scores (0–5) among patients with MIS, this variable was retained as a continuous measure to preserve significant differences in neurological severity, which would be masked if it were dichotomized. Due to the strong correlation between SBP and DBP, only SBP was retained in the multivariable analysis as the representative blood pressure parameter to avoid multicollinearity and improve model stability.

Multivariate logistic regression analysis identified 6 independent factors associated with 90-day unfavorable outcomes after IVT treatment: renal dysfunction (OR = 3.19; 95% CI, 1.94–5.25; *p* < 0.001), NIHSS score (OR = 1.23; 95% CI, 1.07–1.42; *p* = 0.004), functional disability (OR = 2.03; 95% CI, 1.33–3.12; *p* = 0.001), SBP ≥ 140 mmHg (OR = 2.66; 95% CI, 1.71–4.16; *p* < 0.001), neutrophil counts ≥ 6.0 × 10^9^/L (OR = 2.32; 95% CI, 1.60–3.38; *p* < 0.001) and INR ≥ 1.0 (OR = 2.27; 95% CI, 1.56–3.29; *p* < 0.001). Hypertension (OR = 1.03; 95% CI, 0.69–1.52; *p* = 0.895) and FBG ≥ 7.0 mmol/L (OR = 1.43; 95% CI, 0.98–2.07; *p* = 0.062) were excluded due to non-significant associations (Table 3). These 6 predictors were subsequently incorporated into the SPRIN_2_D nomogram, whose corresponding regression coefficients and model intercept were as follows: intercept (−4.058), NIHSS score (0.217), functional disability (0.699), SBP ≥ 140 mmHg (0.982), neutrophil counts ≥ 6.0 × 10^9^/L (0.839), INR ≥ 1.0 (0.817), renal dysfunction (1.172).

**Table 3:** Multivariate logistic regression analysis for 90-day unfavorable outcomes in the derivation cohort.

Predictors	Odds ratio	95% Confidence interval	*p* value
Hypertension			
No			
Yes	1.03	0.69–1.52	0.895
Renal dysfunction			
No			
Yes	3.19	1.94–5.25	<0.001^*^
NIHSS score	1.23	1.07–1.42	0.004^*^
Functional disability			
No			
Yes	2.03	1.33–3.12	0.001^*^
SBP, mmHg			
≥140	2.66	1.71–4.16	<0.001^*^
<140			
FBG, mmol/L			
≥7.0	1.43	0.98–2.07	0.062
<7.0			
Neutrophil count, ×10^9^/L			
≥6.0	2.32	1.60–3.38	<0.001^*^
<6.0			
INR			
≥1.0	2.27	1.56–3.29	<0.001^*^
<1.0			

NIHSS, National Institutes of Health Stroke Scale; SBP, systolic blood pressure; FBG, fasting blood glucose; INR, international normalized ratio. *p < 0.05.

### 3.3 The SPRIN_2_D nomogram

**Figure 2:**
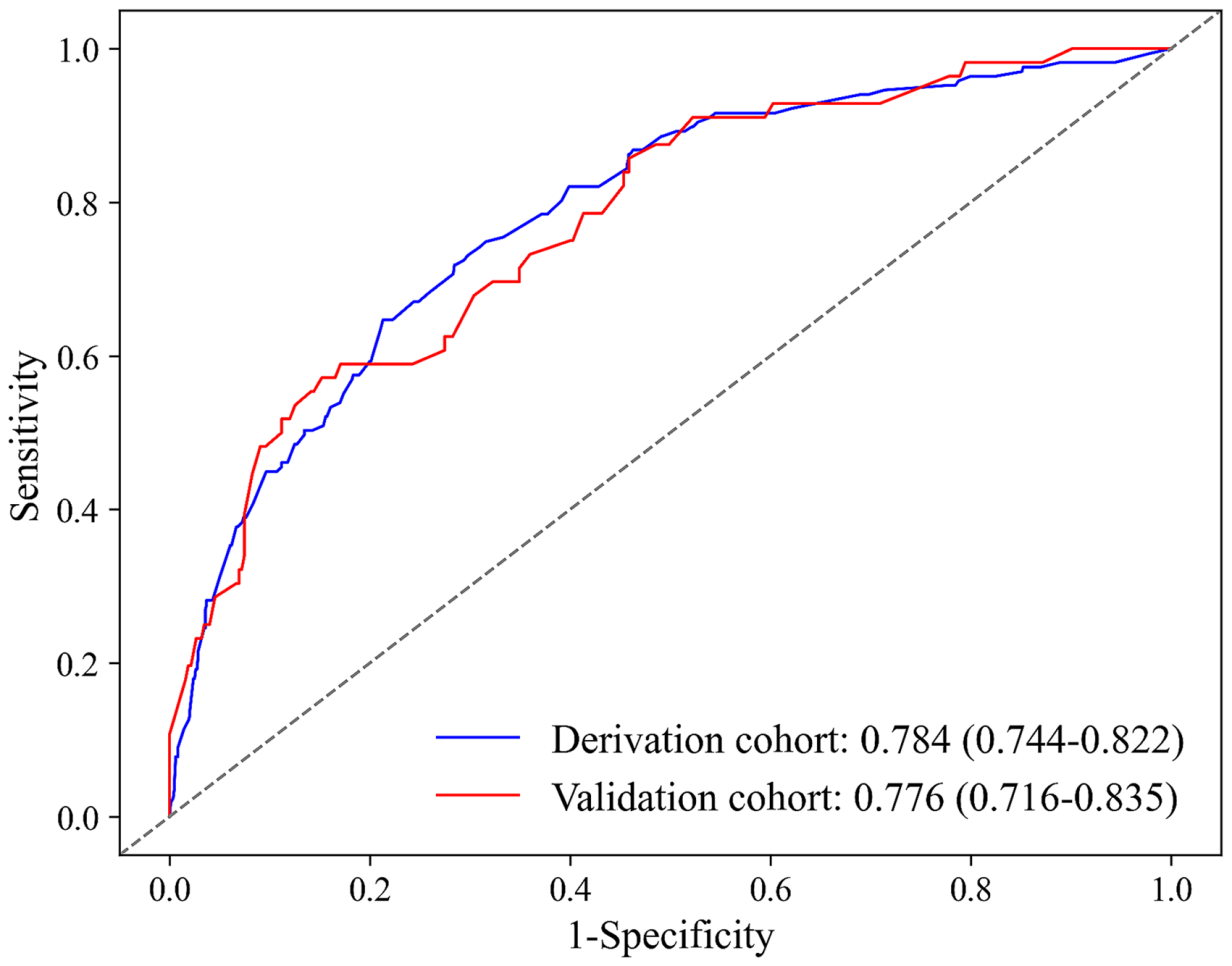
The receiver operating characteristic curve of the SPRIN_2_D nomogram to predict 90-day unfavorable outcomes. Area under curve of derivation cohort and validation cohort was 0.784 (95% confidence interval, 0.744–0.822) and 0.776 (95% confidence interval, 0.716–0.835), respectively.

The SPRIN_2_D nomogram was constructed based on the 6 independent prognostic factors identified in the derivation cohort. The ROC curve analysis suggested that the model exhibited good discriminative ability in both the derivation cohort (AUC = 0.784; 95% CI, 0.744–0.822) and the validation cohort (AUC = 0.776; 95% CI, 0.716–0.835; Figure 2).

**Figure 3:**
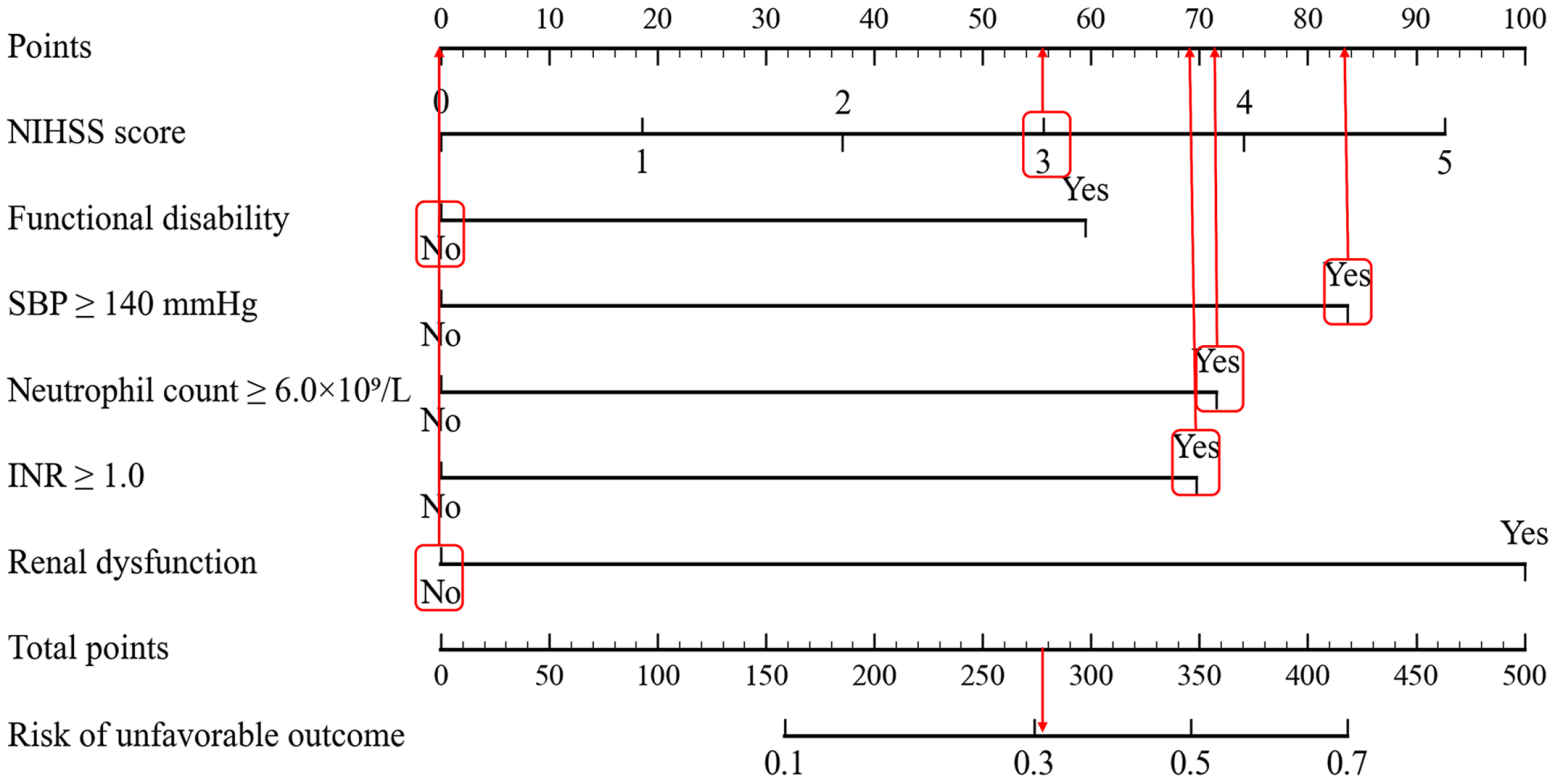
The SPRIN_2_D nomogram. For patients with minor ischemic stroke treated with intravenous thrombolysis, total scores are summed across all individual predictors, and the aggregate total points corresponded to the predicted risk of 90-day unfavorable outcomes. INR, International normalized ratio; SBP, Systolic blood pressure; NIHSS, National Institutes of Health Stroke Scale score.

The nomogram uses a cumulative scoring method: for each patient, the corresponding score for each of the 6 variables is obtained by vertically aligning the variable value with the topmost score axis (total score range: 0–100). The individual scores for all variables are summed to obtain a total score, which is then mapped to the “Total points” axis. A higher total score indicates an increased probability of 90-day unfavorable outcomes. For illustration, a patient with a NIHSS score of 3 (55.5 points), no functional disability (0 points), SBP of 150 mmHg (83.5 points), neutrophil count of 7.5 × 10^9^/L (71.5 points), INR of 1.22 (69 points), and no renal dysfunction (0 points) achieved a total score of 279.5, corresponding to a predicted 90-day unfavorable outcome probability of approximately 31.7% (Figure 3).

Calibration curves were plotted to evaluate the consistency between the predicted and observed probabilities. The SPRIN_2_D nomogram showed good calibration in the derivation cohort (Hosmer-Lemeshow test *p* = 0.289; intercept = −0.001; slope = 1.000; Brier score = 0.115) and the validation cohort (Hosmer-Lemeshow test *p* = 0.196; intercept = −0.218; slope = 1.056; Brier score = 0.096). Non-significant Hosmer-Lemeshow test results, intercepts near 0, slopes close to 1, and low Brier scores collectively confirmed satisfactory consistency between predicted and actual risks (Supplementary Figure S1).

Furthermore, the DCA curve was constructed with threshold probability on the x-axis and net benefit on the y-axis. The SPRIN_2_D nomogram yielded superior net benefit relative to the treat-all and treat-none approaches across threshold ranges of 0.120–0.820 (derivation cohort) and 0.188–0.820 (validation cohort). For patients with elevated predicted risk within these intervals, clinicians can implement intensified surveillance, personalized management and early preventive interventions. Collectively, DCA verified the favorable predictive performance of the SPRIN_2_D nomogram model for 90-day unfavorable outcomes (Supplementary Figure S2).

**Figure 4:**
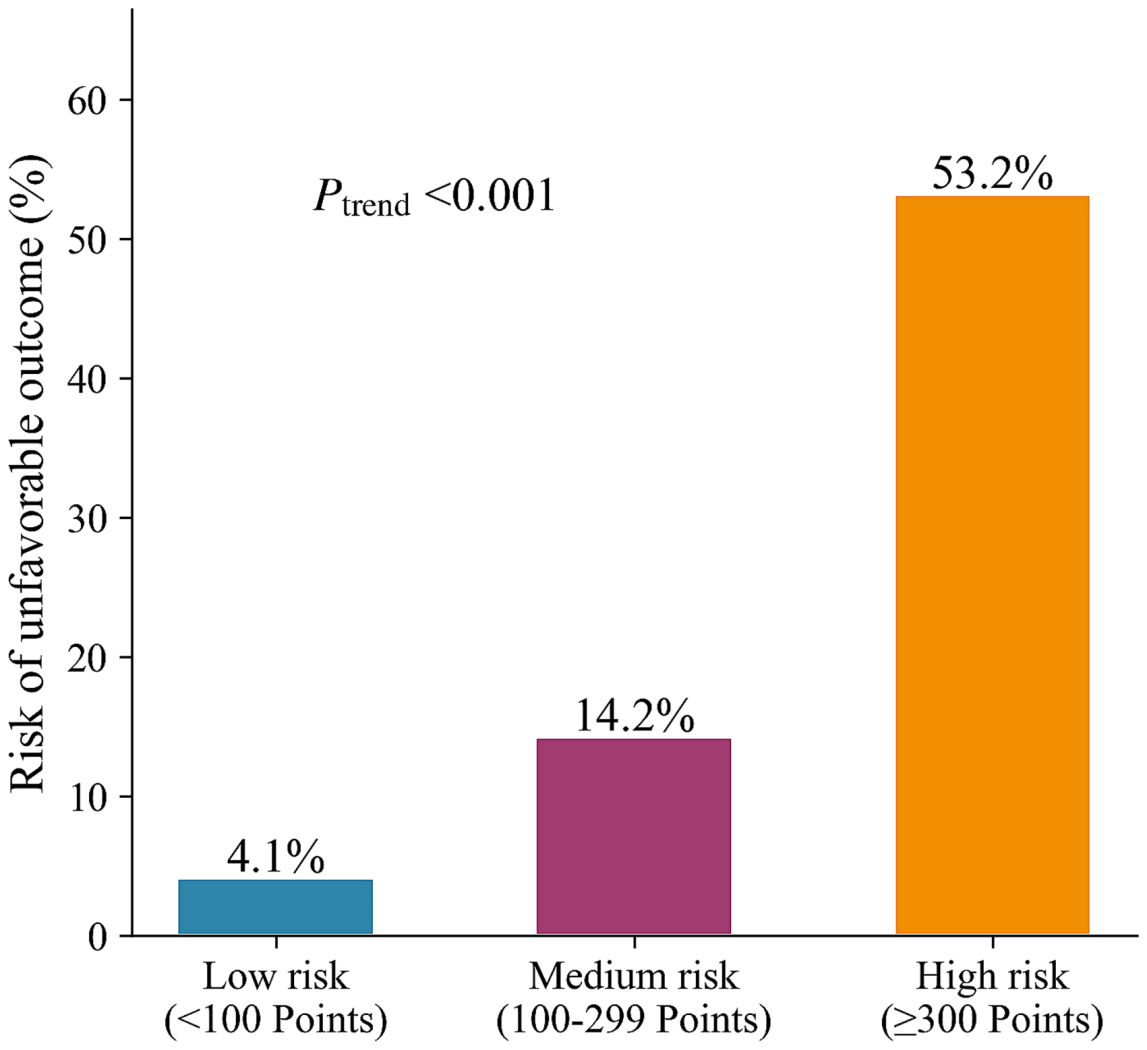
Rates of 90-day unfavorable outcomes stratified by low, medium and high risk groups defined by the SPRIN_2_D nomogram in the derivation cohort.

Subsequently, we evaluated the nomogram’s diagnostic performance across various score thresholds (Table 4). Using total nomogram scores, participants were stratified into low-risk (< 100 points), medium-risk (100–299 points), and high-risk (≥ 300 points) subgroups to facilitate clinical implementation. As summarized in Table 5 and Figure 4, the incidence of 90-day unfavorable outcomes rose substantially from 4.1% (8/194) in the low-risk group to 14.2% (100/702) in the medium-risk group and 53.2% (59/111) in the high-risk group, with a statistically significant increasing linear trend (*p* < 0.001). A consistent progressive risk gradient was also identified in the validation cohort (Supplementary Table S2). Such risk stratification facilitates intuitive nomogram interpretation and helps clinicians readily distinguish patients at varying prognostic risks.

**Table 4:** Diagnostic accuracy of the SPRIN_2_D nomogram for predicting 90-day unfavorable outcomes in derivation cohort.

Score	Proportion of patients (%)	Sensitivity (95% CI)	Specificity (95% CI)	PPV (95% CI)	NPV (95% CI)	PLR (95% CI)	NLR (95% CI)
≥50	92.00%	98.20 (94.85–99.39)	9.29 (7.50–11.44)	17.71 (15.39–20.30)	96.30 (89.67–98.73)	1.08 (1.05–1.12)	0.19 (0.06–0.43)
≥100	80.70%	95.21 (90.83–97.55)	22.14 (19.47–25.07)	19.56 (16.98–22.42)	95.88 (92.08–97.90)	1.22 (1.16–1.29)	0.22 (0.11–0.43)
≥150	58.40%	89.82 (84.30–93.55)	47.86 (44.50–51.24)	25.51 (22.15–29.18)	95.94 (93.60–97.45)	1.72 (1.59–1.87)	0.21 (0.13–0.34)
≥200	36.90%	73.05 (65.86–79.21)	70.24 (67.06–73.23)	32.80 (28.22–37.72)	92.91 (90.65–94.66)	2.45 (2.14–2.82)	0.38 (0.30–0.49)
≥250	20.30%	50.30 (42.80–57.79)	85.71 (83.19–87.92)	41.18 (34.65–48.03)	89.66 (87.37–91.58)	3.52 (2.81–4.40)	0.58 (0.50–0.68)
≥300	11.00%	35.33 (28.48–42.83)	93.81 (91.97–95.25)	53.15 (43.92–62.17)	87.95 (85.65–89.92)	5.71 (4.09–7.97)	0.69 (0.62–0.77)
≥350	5.00%	17.96 (12.88–24.49)	97.62 (96.35–98.45)	60.00 (46.18–72.39)	85.68 (83.32–87.76)	7.54 (4.39–12.96)	0.84 (0.78–0.90)
≥400	1.70%	7.19 (4.16–12.14)	99.40 (98.61–99.75)	70.59 (46.87–87.72)	84.34 (81.95–86.47)	12.07 (4.31–33.81)	0.93 (0.89–0.97)

CI, confidence interval; PPV, positive predictive value; NPV, negative predictive value; PLR, positive likelihood ratio; NLR, negative likelihood ratio.

**Table 5:** Rates of 90-day unfavorable outcomes stratified by SPRIN_2_D-derived risk subgroups in derivation cohort.

SPRIN_2_D risk categories	Unfavorable outcomes (No. of patients)	Total (No. of patients)	Risk (%)	95% Confidence interval	*p* (trend)
Low (<100 points)	8	194	4.1%	2.1–7.9	<0.001^*^
Medium (100–299 points)	100	702	14.2%	11.9–17.0	
High (≥300 points)	59	111	53.2%	43.9–62.2	
Total	167	1,007	—		

*p < 0.05.

To further explore the model’s applicability across distinct stroke subtypes, we excluded 65 patients with undetermined stroke territories, leaving 1,373 eligible participants categorized into anterior circulation stroke (*n* = 1,113) and posterior circulation stroke subgroups (*n* = 260). Subgroup validation via ROC analysis produced an AUC of 0.779 (95% CI, 0.742–0.817) for anterior circulation stroke and 0.765 (95% CI, 0.681–0.843) for posterior circulation stroke (Supplementary Figure S3). These results imply favorable predictive efficacy of the nomogram for posterior circulation stroke. Although the underlying mechanisms responsible for this discrepancy remain unclear, our findings provide supportive evidence regarding this unaddressed question. Additional large-scale cohort validation is required, particularly for the smaller posterior circulation subgroup.

### 3.4 Comparing the SPRIN_2_D nomogram with existing predictive models and individual predictors

**Figure 5:**
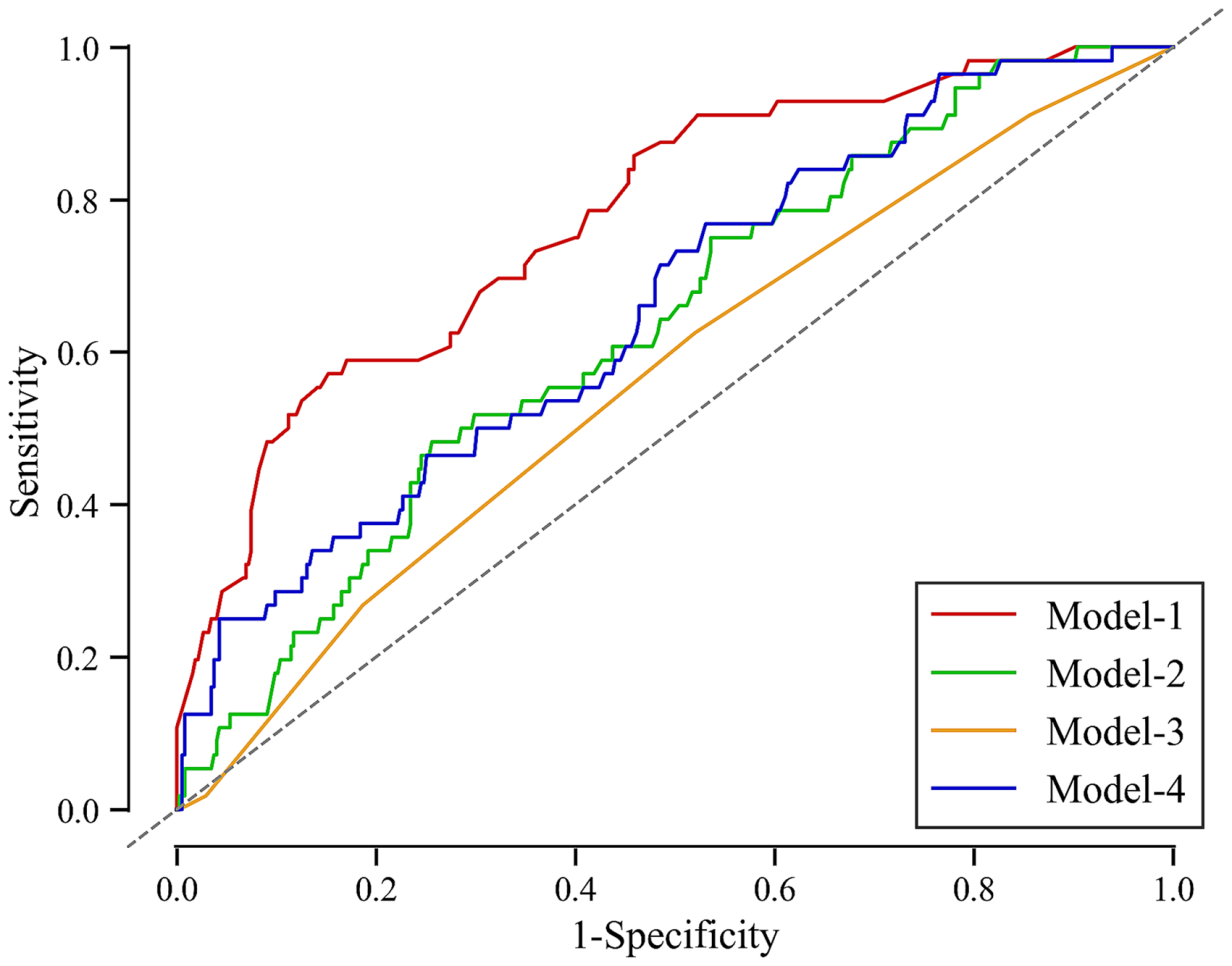
The receiver operating characteristic curves comparing the SPRIN_2_D nomogram with three established prognostic tools for predicting 90-day unfavorable outcomes in the validation cohort. Higher area under curve values correspond to superior discriminative performance. Model-1, SPRIN_2_D nomogram; Model-2, MIS nomogram constructed by Lei et al.; Model-3, THRIVE score; Model-4, Stroke-TPI score.

To further validate the discriminative performance of the SPRIN_2_D nomogram, we compared its predictive performance with that of three published prognostic tools (the THRIVE score, Stroke-TPI score, and Lei’s MIS nomogram) within the validation cohort (*n* = 431) (22–24). Supplementary Table S3 summarizing the key characteristics of the compared models. For 90-day unfavorable outcomes, the corresponding AUC value was 0.631 (95% CI, 0.584–0.677) for the MIS nomogram, 0.567 (95% CI, 0.519–0.614) for the THRIVE score, 0.656 (95% CI, 0.609–0.701) for the Stroke-TPI score, and 0.776 (95% CI, 0.716–0.835) for the SPRIN_2_D nomogram (Figure 5; Table 6). The SPRIN_2_D nomogram yielded the largest AUC value across all assessed models.

**Table 6:** Comparison of area under curves among SPRIN_2_D nomogram and other predictive models for 90-day unfavorable outcomes in validation cohort.

Variables	Area under curve	95% Confidence interval	*p* value
SPRIN_2_D nomogram	0.776	0.716–0.835	-
MIS nomogram	0.631	0.584–0.677	< 0.001^*^
THRIVE score	0.567	0.519–0.614	< 0.001^*^
Stroke-TPI score	0.656	0.609–0.701	0.004^*^

*p < 0.05.

Model calibration was quantified using calibration intercept, slope, and Brier score. The SPRIN_2_D nomogram had a calibration intercept of −0.218 and a slope of 1.056, close to ideal values of 0 and 1, alongside a low Brier score of 0.096 relative to competing models, suggesting minimal prediction error and well-balanced calibration (Supplementary Figure S4; Supplementary Table S4). DCA further confirmed superior clinical utility, as the SPRIN_2_D nomogram generated greater net benefit than the three alternative models over the threshold probability interval of 0.188–0.820 (Supplementary Figure S5).

In the overall cohort (n = 1,438), the AUC values of individual independent predictors ranged from 0.590 to 0.667 (Supplementary Table S5). SBP ≥ 140 mmHg yielded the lowest AUC of 0.590, followed by renal dysfunction (0.591), neutrophil count ≥ 6.0 × 10^9^/L (0.607), INR ≥ 1.0 (0.617), functional disability (0.643), and NIHSS score (0.667). In contrast, the SPRIN_2_D nomogram attained an AUC of 0.779, indicating superior discriminative performance than all standalone predictors.

## 4 Discussion

In clinical practice, over 50% of patients with MIS do not receive IVT treatment, primarily due to their non-disabling symptoms, uncertainty regarding thrombolytic benefit, and concerns about hemorrhagic risk (25). Whether IVT improves outcomes in MIS remains a critical, unresolved clinical question. Accumulating evidence suggests that stroke is a systemic disease with complex pathophysiology, affecting not only the nervous system but also multiple distal organs (26). To address this gap and predict the risk of unfavorable outcomes following thrombolysis in patients with MIS, we developed a nomogram model based on logistic regression analysis. The results identified 6 key predictors of unfavorable outcomes: admission NIHSS score, functional disability, admission SBP ≥ 140 mmHg, INR ≥ 1.0, neutrophil count ≥ 6.0 × 10^9^/L and concurrent renal dysfunction. Validation of the model confirmed its robust discriminative ability and high accuracy.

The admission NIHSS score is a well-established indicator of post-stroke neurological damage and prognosis. It is widely accepted that higher NIHSS scores correlate with an greater likelihood of unfavorable outcomes (27). Findings from the SOCRATES trial further verified the NIHSS score as an independent predictor of subsequent disability in patients with MIS (28). Leira et al. reported an inverse association between baseline NIHSS score (0–6) and 90-day unfavorable outcomes in MIS (29). Similarly, previous studies have linked baseline NIHSS score to IVT efficacy in MIS patients (30). However, the NIHSS score focuses primarily on motor and language function, with limited assessment of higher-level neurological functions. After eliminating multicollinearity, we incorporated functional disability into the model to overcome this limitation, which can comprehensively reflect the severity of stroke. A previous analysis showed that disabled AIS patients treated with IVT were more likely to recover to their pre-stroke functional status (31), supporting the clinical relevance of this variable. Nevertheless, as the definition of functional disability is partially constructed from individual NIHSS items, potential overlapping information between total NIHSS scores and this functional disability metric merits careful consideration. In our predictive model, the total NIHSS score reflects the overall severity of neurological impairment, whereas the functional disability variable specifically captures persistent disabling manifestations that interfere with patients’ daily functional capacity. Accordingly, the independent prognostic value of functional disability should be interpreted against this clinical context.

In recent years, the critical role of inflammation in stroke pathophysiology has been increasingly recognized. After stroke, neutrophils infiltrate the central nervous system, releasing matrix metalloproteinase-9, reactive oxygen species, and other proteases that induce further blood–brain barrier damage and impede vascular recanalization (32). Thrombus analysis in AIS patients revealed that neutrophil content was significantly correlated with thrombolysis resistance (33), and the pre-thrombolysis neutrophil count is an independent predictor of 90-day unfavorable outcomes in AIS patients (34). The neutrophil-to-lymphocyte ratio, which is derived from neutrophils, has also been shown to be associated with END following IVT (35). Furthermore, increased neutrophil counts and ratios have also been associated with an elevated risk of stroke recurrence in patients with MIS (36). Consistent with these findings, our study confirmed a significant correlation between neutrophil counts and 90-day unfavorable outcomes in MIS patients receiving IVT therapy, highlighting the prognostic value of inflammatory markers.

Inflammation and the coagulation system exhibit extensive crosstalk across multiple stages of stroke pathology (37). Earlier research focused on prothrombin time (PT), but INR—an adjusted index of PT—can more accurately predict unfavorable outcomes of patients with AIS by adjusting the PT ratio and laboratory-specific differences (38). Elevated admission INR has been linked to unfavorable functional outcomes in Chinese AIS patients and to END in AIS patients undergoing (38, 39). Although the underlying mechanism remains unclear, researchers have hypothesized that decreased coagulation factors, an enhanced inflammatory response, and increased bleeding risk collectively contribute to this outcome (37, 40). Our findings further emphasize the prognostic significance of INR in MIS treatment.

Renal dysfunction was also identified as an independent predictor. A meta-analysis illustrated the connection between renal dysfunction and increased stroke incidence in the general population, attributed to shared pathogenic mechanisms including vascular damage and endothelial injury (41). In particular, evidence exhibited that AIS patients with renal insufficiency have higher rates of disability and hemorrhagic transformation after IVT therapy (42), partly due to impaired endothelial tPA release and reduced fibrinolytic activity (43). Additionally, platelet and coagulation abnormalities associated with renal insufficiency increase bleeding risk following thrombolysis (44), justifying the inclusion of renal dysfunction in our nomogram.

Finally, elevated admission SBP, a traditional stroke prognostic factor, was incorporated into the nomogram. High SBP reflects abnormal vascular autoregulation, and wider blood pressure fluctuations after IVT treatment aggravate reperfusion injury to the blood–brain barrier, leading to severe complications such as cerebral edema and intracranial hemorrhage (45, 46). The ENCHANTED trial further indicated that lower SBP levels are associated with better outcomes in AIS patients receiving IVT treatment (47). In addition, validation of our model in anterior and posterior circulation stroke subgroups provides deeper insights into the study of MIS, with superior performance observed in posterior circulation stroke patients—though the underlying mechanism requires further investigation.

Admission FBG was significantly correlated with 90-day unfavorable outcomes in univariate analysis but lost independent prognostic value after multivariate adjustment and was not incorporated into the final SPRIN_2_D nomogram. Multiple prior investigations have validated admission hyperglycemia as an unfavorable prognostic indicator for AIS, which exacerbates cerebrovascular injury via vascular dysfunction and oxidative stress-triggered pathological processes (48, 49). Accumulated clinical evidence further demonstrates that elevated admission glucose is closely associated with aggravated neurological impairment, increased in-hospital mortality and a higher risk of END in heterogeneous AIS populations (50, 51). Nevertheless, this independent prognostic effect of admission hyperglycemia was absent in our MIS cohort. Such inconsistency is likely attributable to population characteristics: all included patients suffered only mild neurological deficits and fewer systemic comorbidities, thereby attenuating the independent prognostic potency of admission FBG. Further large-scale multicenter prospective trials adopting standardized blood glucose testing protocols are warranted to verify our results.

Clinically, the SPRIN_2_D nomogram was developed for risk stratification rather than treatment decision-making. It should not be used to determine eligibility for IVT, which must be prescribed in accordance with established clinical criteria and current guidelines. Instead, identifying patients at high risk of unfavorable outcomes enables clinicians to conduct closer neurological monitoring, formulate targeted rehabilitation plans, and deliver individualized post-discharge care. Notably, patients in the low-risk group should receive standard care, rehabilitation, and secondary prevention as recommended by guidelines. Collectively, this nomogram mainly serves as a tool for early risk evaluation and optimization of post-thrombolysis management.

Compared with existing prediction models, the SPRIN_2_D nomogram has several strengths. Firstly, the model was derived from a large, multicenter cohort, enhancing the generalizability of the findings. Secondly, all independent predictors can be quickly obtained before thrombolysis, facilitating individualized follow-up planning. Thirdly, these variables are widely available in primary hospitals, enabling broad implementation of the nomogram. Fourthly, the study enrolled both disabled and non-disabled patients with MIS receiving IVT therapy, ensuring greater representativeness and providing a more comprehensive reference for MIS prognosis.

Nonetheless, this study has certain limitations. (1) Participating hospitals in the TRAIS study were municipal hospitals with relatively advanced medical resources, potentially introducing selection bias that may limit the generalizability of the results to resource-limited settings. (2) The high prevalence of mRS scores of 0–1 at 90 days among MIS patients may lead to “ceiling effects,” potentially influencing outcome assessment (52). Future studies based on larger multicenter cohorts may adopt alternative outcome definitions, such as mRS 1–6 and ordinal outcome models, to evaluate their incremental prognostic value and further improve model performance. (3) Laboratory variables may fluctuate during hospitalization, and future studies should consider dynamic monitoring to capture temporal changes in these markers. (4) Key clinical metrics including thrombolytic dose and door-to-needle time were not incorporated into the regression analysis, possibly resulting in omitted variable bias and compromised model robustness. Well-designed prospective cohorts adopting standardized data collection protocols are warranted to integrate these covariates and improve the nomogram’s clinical practicability. (5) While clinically meaningful categorization improves the practicality and interpretability of the model, it inevitably leads to information loss from the original continuous variables. Accordingly, larger multicenter cohorts are needed to validate our results. Further studies could also adopt continuous-variable approaches, such as restricted cubic splines, to explore whether these methods can enhance model calibration, discrimination and clinical utility. (6) Independent external validation across geographically and clinically heterogeneous cohorts is required prior to widespread clinical adoption of the SPRIN_2_D nomogram.

## 5 Conclusion

We successfully constructed and validated the SPRIN_2_D nomogram, a stable clinical tool designed to predict 90-day unfavorable outcomes in patients with MIS undergoing IVT treatment. As a user-friendly and accessible tool, the SPRIN_2_D nomogram addresses the clinical gap caused by the lack of insufficient MIS-specific prognostic models, offering clinicians actionable risk stratification to support individualized follow-up planning. It also contributes to the efficient utilization of medical resources by identifying high-risk patients for closer monitoring and targeted interventions, thereby advancing the clinical management of MIS.

## Data Availability

The original contributions presented in the study are included in the article/Supplementary material, further inquiries can be directed to the corresponding authors.

## Glossary

TRAIS: Multicenter Clinical Trial of Revascularization Treatment for Acute Ischemic Stroke
AIS: Acute Ischemic Stroke
MIS: Minor Ischemic Stroke
END: Early Neurological Deterioration
IVT: Intravenous Thrombolysis
NIHSS: National Institutes of Health Stroke Scale
FBG: Fasting Blood Glucose
GFR: Glomerular Filtration Rate
SBP: Systolic Blood Pressure
DBP: Diastolic Blood Pressure
PT: Prothrombin Time
APTT: Activated Partial Thromboplastin Time
INR: International Normalized Ratio
mRS: modified Rankin Scale
SD: Standard Deviation
IQR: Interquartile Range
OR: Odds Ratio
ROC: Receiver Operating Characteristic
AUC: Area Under the Curve
DCA: Decision Curve Analysis
tPA: tissue Plasminogen Activator
CI: Confidence Interval
VIF: Variance Inflation Factor
NLR: Negative Likelihood Ratio
NPV: Negative Predictive Value
PLR: Positive Likelihood Ratio
PPV: Positive Predictive Value
