# Supplementary material for: SPRIN2D nomogram: predicting 90-day unfavorable outcomes in patients with minor ischemic stroke receiving intravenous thrombolysis
Source: Front Neurol. 2026 Aug 3;17:1841196. doi: 10.3389/fneur.2026.1841196 (PMC13477958; doi:10.3389/fneur.2026.1841196)
Supplement: Supplementary file 1 [file Data_Sheet_1.docx]

**Supplementary material**

**Figure S1** Calibration curves of the SPRIN_2_D nomogram for the derivation (A) and validation (B) cohorts, with predicted probability plotted on the horizontal axis and observed probability on the vertical axis.

**Figure S2** Decision curve analysis for the SPRIN_2_D nomogram across the derivation and validation cohorts.

**Figure S3** Receiver operating characteristic-derived area under the curve values of the SPRIN_2_D nomogram for patients with anterior and posterior circulation stroke, at 0.779 (95% CI, 0.742-0.817) and 0.765 (95% CI, 0.681-0.843), respectively.

**Figure S4** Calibration curves comparing the SPRIN_2_D nomogram against established acute ischemic stroke prognostic models for 90-day unfavorable outcome in the validation cohort. Model-1: SPRIN_2_D nomogram; Model-2: MIS nomogram constructed by Lei et al; Model-3: THRIVE score; Model-4: Stroke-TPI score.

**Figure S5** Decision curve analysis comparing the SPRIN_2_D nomogram and with other existing prognostic tools for predicting 90-day unfavorable outcomes in the validation cohort. Model-1: SPRIN_2_D nomogram; Model-2: MIS nomogram constructed by Lei et al; Model-3: THRIVE score; Model-4: Stroke-TPI score.

**Table S1** Tolerance and variance inflation factor values for variables included in multivariable regression model

| Variables | Tolerance | Variance inflation factor |
| --- | --- | --- |
| NIHSS score | 0.725 | 1.380 |
| Functional disability | 0.727 | 1.375 |
| SBP ≥ 140, mmHg | 0.958 | 1.044 |
| FBG ≥ 7.0, mmol/L | 0.984 | 1.016 |
| Neutrophil count ≥ 6.0×10^9^/L | 0.977 | 1.024 |
| INR ≥ 1.0 | 0.976 | 1.025 |
| Renal dysfunction | 0.956 | 1.046 |
| Hypertension | 0.933 | 1.072 |

**Abbreviations:** NIHSS, National Institutes of Health Stroke Scale; SBP, systolic blood pressure; FBG, fasting blood glucose; INR, international normalized ratio

**Table S2** Rates of 90-day unfavorable outcomes stratified by SPRIN_2_D-derived risk subgroups in validation cohort

| SPRIN_2_D risk categories | Unfavorable outcomes  (No. of patients) | Total  (No. of patients) | Risk (%) | 95% CI | *p* (trend) |
| --- | --- | --- | --- | --- | --- |
| Low (<100 points) | 2 | 85 | 2.4% | 0.6%-8.2% | <0.001^*^ |
| Medium (100-299 points) | 36 | 301 | 12.0% | 8.8%-16.1% |  |
| High (≥300 points) | 18 | 45 | 40.0% | 27.0%-54.5% |  |
| Total | 56 | 431 | — |  |  |

Abbreviation: CI, confidence interval. **p* < 0.05

**Table S3** Characteristics and originally reported discriminative performance of the SPRIN_2_D nomogram and existing prognostic models for acute ischemic stroke

| Models | Predictors | Target population | Outcome definition | Original reported AUC value |
| --- | --- | --- | --- | --- |
| SPRIN_2_D nomogram | Baseline NIHSS score; functional disability; systolic blood pressure; neutrophil count; international normalized ratio; Renal dysfunction | Patients with minor ischemic stroke receiving intravenous thrombolysis | 90 day unfavourable outcomes (mRS 2-6) | AUC = 0.784  (derivation cohort) |
| MIS nomogram | Gender; age; baseline NIHSS score; hypertension; diabetes mellitus; white blood cell; serum uric acid; | Patients with minor stroke and transient ischemic attack | 90 day unfavourable outcomes (mRS 2-6) | AUC = 0.775  (derivation cohort) |
| THRIVE score | Age; NIHSS; hypertension; diabetes mellitus; atrial fibrillation | Patients with ischemic stroke receiving intravenous thrombolysis | 90 day unfavourable functional outcomes (mRS 3-6) | AUC = 0.756  (overall study development dataset) |
| Stroke-TPI score | Age; sex; diabetes mellitus; baseline NIHSS score; systolic blood pressure; previous stroke; time from symptom onset to treatment; rt-PA treatment | Patients with acute ischemic stroke | 90 day favourable functional outcome: (mRS 0-1) | AUC = 0.788  (overall study development dataset) |

**Abbreviations:** NIHSS, National Institutes of Health Stroke Scale; mRS, modified rankin scale; AUC, area under receiver

**Table S4** Calibration performance of the SPRIN_2_D nomogram and established acute ischemic stroke prognostic models for 90-day unfavorable outcome prediction in the validation cohort

| Models | *p* value of the H-L test | Intercept | Slope | Brier score |
| --- | --- | --- | --- | --- |
| SPRIN_2_D nomogram  nomogram | 0.196 | -0.218 | 1.056 | 0.096 |
| MIS nomogram | <0.001 | -1.403 | 0.445 | 0.143 |
| THRIVE score | 0.081 | -0.011 | 0.993 | 0.113 |
| Stroke-TPI score | 0.201 | -0.293 | 1.016 | 0.109 |

**Abbreviation:** H-L test, Hosmer-Lemeshow test

**Table S5** Comparison of predictive performance between individual predictors and the SPRIN_2_D nomogram across the full study cohort

| Variables | AUC (95% CI) | Sensitivity (95% CI) | Specificity (95% CI) |
| --- | --- | --- | --- |
| NIHSS score | 0.667 (0.628-0.706) | 0.750 (0.725-0.774) | 0.520 (0.455-0.586) |
| Functional disability | 0.643 (0.608-0.678) | 0.756 (0.732-0.781) | 0.529 (0.464-0.595) |
| SBP ≥ 140 mmHg | 0.590 (0.560-0.620) | 0.386 (0.359-0.413) | 0.794 (0.741-0.847) |
| Neutrophil count ≥ 6.0×10^9^/L | 0.607 (0.572-0.642) | 0.744 (0.719-0.769) | 0.471 (0.405-0.536) |
| INR ≥ 1.0 | 0.617 (0.583-0.652) | 0.612 (0.584-0.639) | 0.623 (0.560-0.687) |
| Renal dysfunction | 0.591 (0.562-0.620) | 0.936 (0.922-0.950) | 0.247 (0.190-0.303) |
| SPRIN_2_D nomogram | 0.779 (0.746-0.812) | 0.776 (0.721-0.831) | 0.639 (0.612-0.666) |

**Abbreviations:** AUC, area under receiver; CI, confidence interval; NIHSS, National Institutes of Health Stroke Scale; SBP, systolic blood pressure; INR, international normalized rati

**Figure S1**

**Figure S2**

**Figure S3**

**Figure S4**

**Figure S5**
